# Arthroscopic screw versus suture fixation in tibial eminence fractures: a systematic review and meta-analysis

**DOI:** 10.1186/s43019-025-00282-5

**Published:** 2025-07-24

**Authors:** Fadlurrahman Manaf, Lukas Widhiyanto, Kukuh Dwiputra Hernugrahanto

**Affiliations:** 1 Department of Orthopaedics and Traumatology, Faculty of Medicine, Universitas Airlangga, Mayjend. Prof. Dr. Moestopo No 6-8, Surabaya, 60286 East Java Indonesia; 2 Department of Orthopaedics and Traumatology, Dr. Soetomo General Academic Hospital, Surabaya, Indonesia

**Keywords:** Tibial eminence fracture, Health outcomes, Screws, Suture, Complications

## Abstract

**Background:**

Tibial eminence fractures are common injuries that can cause significant functional limitations and require timely and effective treatment. Arthroscopic screw fixation and suture fixation are the primary methods used for managing displaced fractures. This study aimed to compare the functional and clinical outcomes between the two groups.

**Methods:**

An associated systematic review was carried out with Preferred Reporting Items for Systematic Reviews and Meta-Analyses (PRISMA) guidelines utilizing the PubMed, Cochrane Central Register of Controlled Trials (CENTRAL), Sage Journals, Science Direct, and Core Journals databases. Any language publication that assessed the results following the fixation of tibial eminence fractures by screw and suture fixation from 2000 to 2024 was included. Clinical, functional outcomes, subsequent surgeries, complications, operation time, and union time were evaluated. All data were assessed using SPSS version 25 and RevMan version 5.4.

**Results:**

A total of 9 studies involving 412 patients were analyzed out of 3365 papers. There were no significant differences (*p* > 0.05) between the two methods in the Lysholm score, Tegner Activity Scale, International Knee Documentation Committee (IKDC) score, range of motion, Lachman test, pivot-shift test, KT-1000, and union time. However, screw fixation had a significantly higher rate of subsequent surgeries (planned removal implant excluded) (29.75% versus 11.6%; *p* < 0.00001), complications (*p* = 0.0003), and shorter operation times (67 min versus 85 min; *p* = 0.0003).

**Conclusions:**

The findings revealed that suture fixation carried a significantly lower risk of subsequent surgery and complications but required a longer operation time. Each technique presents advantages and challenges, making the decision a crucial aspect of patient care.

## Background

Anterior cruciate ligament (ACL) avulsion fractures or tibial eminence fractures often occur in young adults. Most cases are caused by sports and traffic accidents [1]. With an incidence rate of 3 per 100,000 cases a year and leading to 2–5% of knee injuries in the pediatric population, it can be said to be a relatively rare fracture [2, 3]. Tibial eminence fractures can cause instability in the knee joint and mechanical blockage due to intra-articular fragments. Therefore, surgical treatment is the main choice of treatment for displaced tibial eminence fracture to restore ACL function as a stabilizer and eliminate mechanical interference when the knee joint moves [4, 5]. In terms of the type of fixation, Kirschner wires, screws, sutures, or suture anchors can be used. The use of metal screws and suture fixation with nonabsorbable threads is more widely used [4, 6]. 

Several studies have evaluated the positive and negative aspects of each fixation type in patients with tibial eminence fractures. According to recent research, during cyclic loading testing, suture fixation with fiber wire offers greater stability than screw fixation [7]. According to a different study, during cyclic loading testing, suture fixation is less stable than screw fixation [8]. Another study noted that the outcomes between the use of screw and suture fixation were not statistically different [4]. The comparative effectiveness of these two methods remains inconclusive. This systematic review and meta-analysis aimed to compare the clinical and functional outcomes of arthroscopic screw and suture fixation in patients with displaced tibial eminence fractures. We hypothesized that there would be no significant difference in the clinical and functional outcomes between the two fixation methods.

## Methods

This systematic review and meta-analysis followed the Preferred Reporting Items for Systematic Reviews and Meta-Analyses (PRISMA) guidelines. A comprehensive search was conducted in databases, including PubMed, Cochrane CENTRAL, Sage Journals, ScienceDirect, and Core Journals, covering all available data up to May 2024. The search keywords were categorized into three domains: (1) case (e.g., “tibial eminence” OR “tibial intercondylar eminence” OR “tibial spine” OR “anterior cruciate ligament avulsion fractures”), (2) intervention (e.g., “screw fixation” OR “suture fixation” OR “arthroscopic”), and (3) outcome (e.g., “clinical outcome” OR “functional outcome”). These categories were combined using Boolean operators in the following search patterns: (1 AND 2), (1 AND 3), and (1 AND 2 AND 3). This review was conducted in accordance with a predefined protocol, which was registered with the International Prospective Register of Systematic Reviews (PROSPERO) under the registration no. CRD42021243548 (https://www.crd.york.ac.uk/PROSPERO/view/CRD42021243548).

The database citations were checked for duplication. The titles and abstracts of the remaining citations were thoroughly screened. This review included studies involving patients with displaced tibial eminence fracture (Meyers and McKeever grades II–IV) who underwent arthroscopic fixation surgery with screw or suture. The primary outcomes were clinical and functional outcomes, including Lysholm score, International Knee Documentation Committee (IKDC) score, Tegner Activity Scale, Lachman test, pivot-shift test, KT-1000 arthrometer measurements, and range of motion. Secondary outcomes included complication rates, subsequent surgeries, operation time, and union time. Studies eligible for inclusion comprised randomized controlled trials, cohort studies, and case–control studies that directly compared screw fixation and suture fixation techniques in the specified patient population. Reviews, study protocols, reports (technical or case), systematic reviews, meta-analyses, case series, studies with incomplete data, animal studies, cadaveric studies, and laboratory studies were excluded. After evaluating the studies, we determined which ones qualified for the analysis. The Risk of Bias in Nonrandomized Studies of Intervention (ROBINS-I) tool was used to evaluate the study’s bias.

Data were extracted from the selected articles, including: (1) clinical outcomes (KT-1000, pivot-shift test, Lachman test, and range of motion); (2) functional outcomes (Tegner Activity Scale, Lysholm score, and IKDC score); (3) patient characteristics (age, sex, fracture type, and follow-up duration); (4) subsequent surgeries; (5) complications; (6) operation time; and (7) union time. Planned implant removal was excluded in analysis of subsequent surgeries. Complication rates, indications for subsequent surgery, and rehabilitation program were also extracted and analyzed.

The Statistical Package for the Social Sciences (SPSS) version 25 and Review Manager version 5.4 software (The Nordic Cochrane Center, The Cochrane Collaboration) were used to conduct this meta-analysis. The Mantel–Haenszel method was used to analyze binary data and to calculate risk ratio with corresponding 95% confidence intervals (CIs). The inverse-variance method was used to analyze continuous data and to calculate mean differences with corresponding 95% CIs. *I*^2^ statistics with 0% and 100% values were used to measure and quantify the heterogeneity. Heterogeneity was shown by an *I*^2^ value greater than 50%, and a random-effects model was used to compare studies. For studies with little to no heterogeneity, a fixed-effects model was employed. Other analyses, such as independent *t*-tests, were used for continuous variables, and chi-squared tests were used for categorical variables. A *p*-value of less than 0.05 was considered statistically significant for all analyses.

## Results

**Fig. 1:**
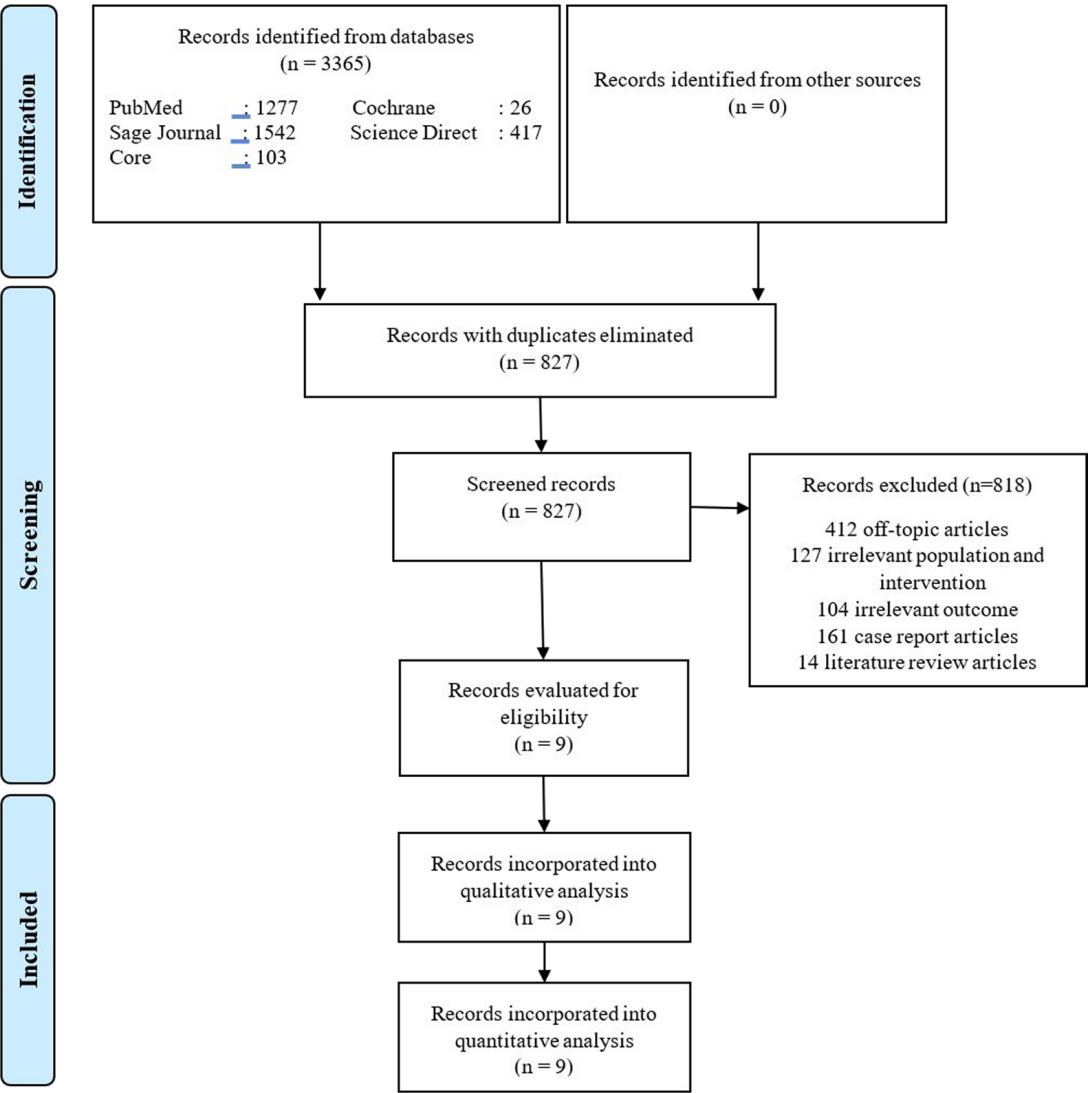
Flow diagram based on PRISMA guidelines

**Fig. 2:**
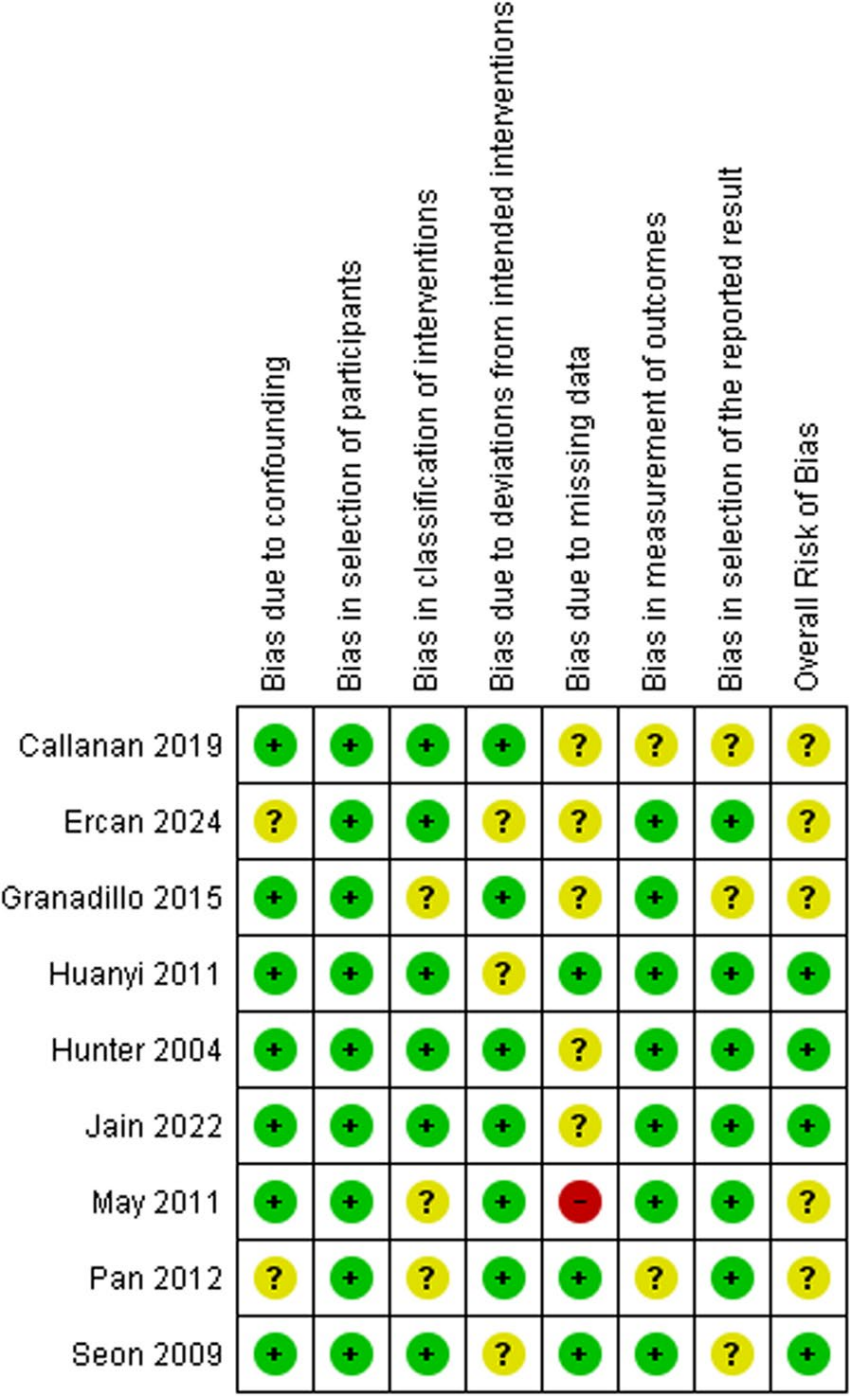
Summary of study risk of bias using ROBINS-I

On the basis of the search results, 3365 potential studies were identified (Fig. 1). Duplicate exclusion was subsequently performed, and 827 articles were obtained. Screening of the 827 studies was carried out on the basis of the study title and abstract. After exclusion, nine studies were obtained and assessed qualitatively and quantitatively. A summary of the risk of bias for each study is provided in Fig. 2. Most studies showed low-to-moderate risk of bias, primarily due to missing data.

The included studies were published between 2004 and 2024, involving a total of 412 patients: 207 in the suture group and 205 in the screw group. Most participants were male, with ages ranging from 6 to 60 years. On average, the ages of the patients in the screw and suture groups were similar, although variations existed between the studies (20.04 ± 9.54 years versus 17.24 ± 6.23 years; *p* = 0.471). All studies focused on patients with Meyers and McKeever classification type II or III fractures, with some including type IV fractures, demonstrating the applicability of both methods to similar fracture types (type II: *p* = 0.288; type III: *p* = 0.301; type IV: *p* = 0.587). The follow-up duration varied widely from 6 to 84 months (48.34 ± 16.27 months versus 41.46 ± 15.24 months; *p* = 0.510). Sex distribution also showed no significant differences (male: *p* = 0.450; female: *p* = 0.479). Baseline characteristics were similar between two groups (Tables 1, 2).

**Table 1:** Data demographics included in this study

Studies	Group	Patients, *n* (M:F)	Age Mean (range), years	Types of fracture, *n* (type II/type III/type IV)	Follow-up Mean (range), months	Conclusion
Hunter (2004)	Screw	9 (1:8)	39.5 (16–60.1)	5/4/–	32.6 (14–51)	No significant difference
	Suture	8 (4:4)	12.375 (7.5–25)	3/5/–		
Seon (2009)	Screw	16 (10:6)	16.8 (10–29)	6/10/–	29.7 (24–36)	No significant difference
	Suture	17 (13:4)	16 (8–34)	5/12/–	29.5 (24–36)	
May (2011)	Screw	6	15.8 (11–29)	3/3/–	84 (24–180)	No significant difference
	Suture	12	16.3 (7–39)	6/6/–		
Huanyi (2011)	Screw	21 (16:5)	22.4 (15–37)	6/15/–	68.4	No significant difference
	Suture	22 (17:5)	23.8 (16–39)	8/14/–	63.6	
Pan (2012)	Screw	25 (14:11)	25 (18–52)	9/15/1	58 (24–100)	Screw is superior
	Suture	23 (16:7)	25 (17–50)	8/14/1	47 (24–93)	
Granadillo (2015)	Screw	35 (24:11)	11.3 ± 2.77	15/20/–	6	No significant difference
	Suture	36 (25:11)	11.7 ± 2.61	16/20/–		
Callanan (2019)	Screw	35 (27:8)	11.2 ± 3.29	9/22/4	51.6	No significant difference
	Suture	33 (22:11)	12.4 ± 2.55	5/28/–	25.2	
Jain (2022)	Screw	45 (32:13)	27.4 (18–42)	15/30/–	9	Suture is superior
	Suture	45 (34:11)	26.62 (18–49)	17/28/–		
Ercan (2024)	Screw	13 (9:4)	11 (6–13)	5/8/–	34 (24–48)	No significant difference
	Suture	11 (8:3)	11 (7–14)	5/6/–	42 (24–60)

**Table 2:** Analysis of demographic data between two groups

	Screw (*n* = 205)	Suture (*n* = 207)	*p*
Age (years), mean ± standard deviation (SD)^†^	20.04 ± 9.54	17.24 ± 6.23	0.471
Sex^*^, *n* (%)^‡^			
Male	133 (66.8)	139 (71.3)	0.450
Female	66 (33.2)	56 (28.7)	0.479
Follow-up^**^, months, mean ± SD^†^	48.34 ± 16.27	41.46 ± 15.24	0.510
Fracture type, *n* (%)^‡^			
Type II	73 (35.9)	73 (34.9)	0.288
Type III	127 (61.6)	133 (64.6)	0.301
Type IV	5 (2.5)	1 (0.5)	0.587

^†^Independent *t*-test

^‡^Chi-squared test

^*^Eight studies included

^**^Five studies included

**Fig. 3:**
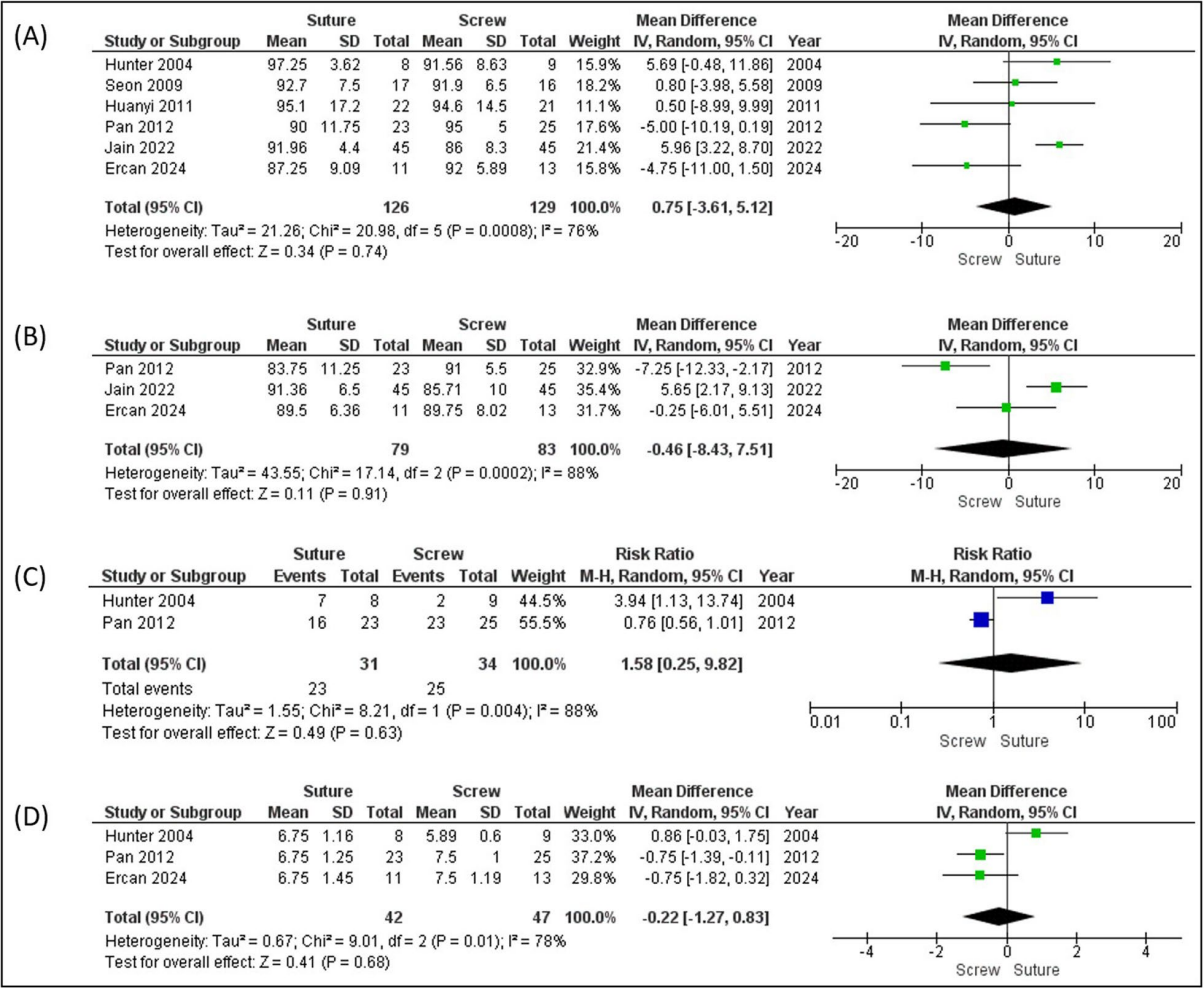
Forest plot analysis. **A** Lysholm score, **B** IKDC subjective score, **C** IKDC objective score, and **D** Tegner Activity Scale

The postoperative Lysholm scores showed no significant difference between the two groups, with scores of 92.37 for the suture group and 91.84 for the screw group (mean difference (MD) 0.75 [−3.61–5.12]; *p* = 0.74) (Fig. 3A). Similarly, the IKDC subjective score between the groups was not statistically significant, with scores of 88.20 and 88.82 for the suture and screw groups, respectively (MD 0.46 [−8.43–7.51]; *p* = 0.91) (Fig. 3B). The IKDC objective scores were 73% and 74% for the screw and suture groups, respectively (relative risk (RR) 1.58 [0.25−9.82]; *p* = 0.63) (Fig. 3C). The Tegner Activity Scale scores were similar between the groups, with the suture group scoring 6.75 and the screw group scoring 6.86 (MD 0.22 [1.27–0.83]; *p* = 0.68), showing no significant difference (Fig. 3D).

**Fig. 4:**
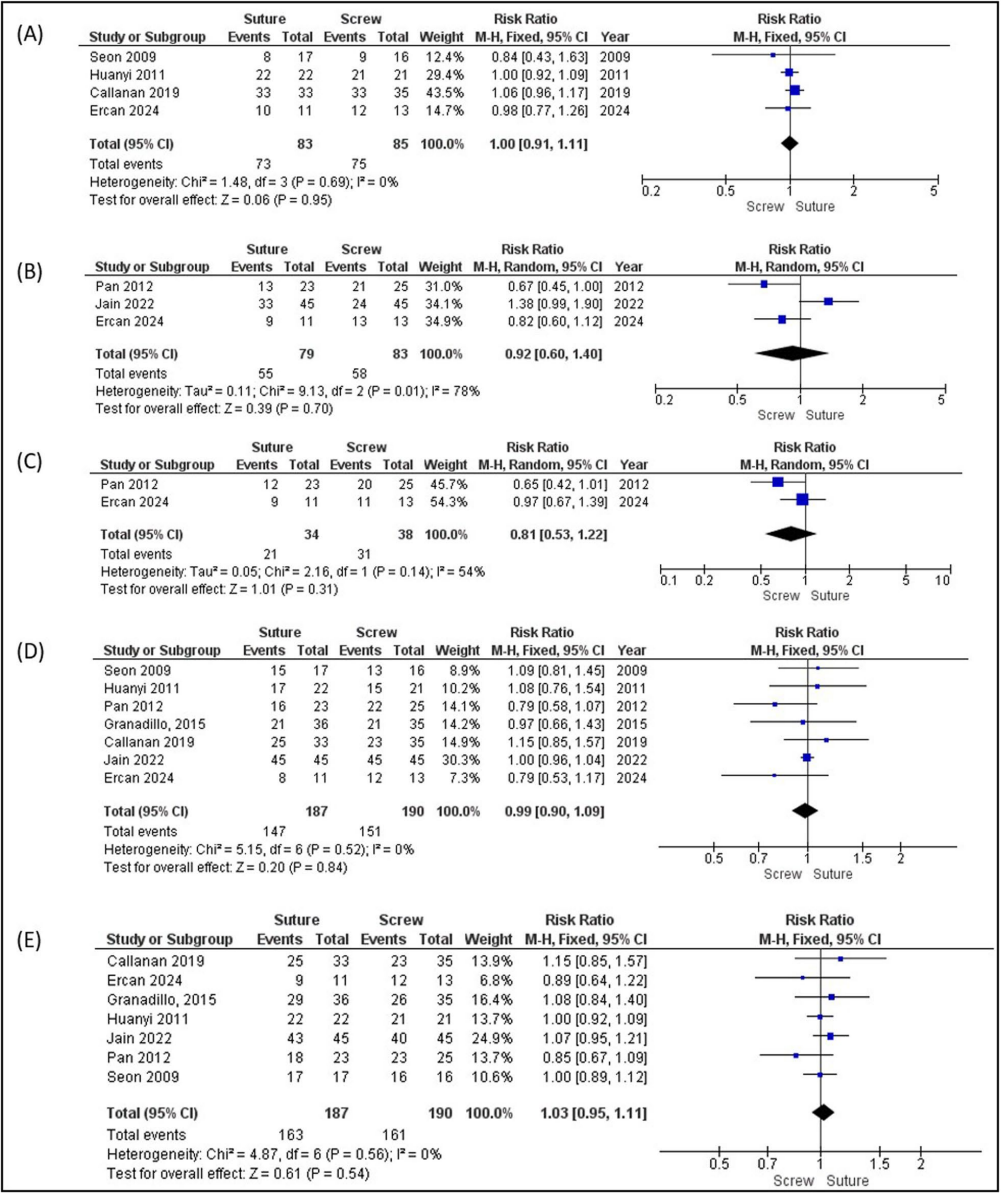
Forest plot analysis. **A** Lachman test assessed as grade 0 or negative, **B** pivot-shift test assessed as grade 0 or negative, **C** KT-1000 assessed as < 3-mm side-to-side differences, **D** lag extension assessed as < 2°, and **E** lag flexion assessed as < 5°

The Lachman test was assessed as a stable knee joint, with 88% in both groups (RR 1.00 [0.91−1.11]; *p* = 0.95) (Fig. 4A). The pivot-shift test results were also assessed as stable at 70% for both groups (RR 0.92 [0.60−1.40]; *p* = 0.70) (Fig. 4B). KT-1000 was assessed as a < 3-mm side-to-side difference, 81% and 62% for the screw and suture groups, respectively (RR 0.81 [0.53−1.22]; *p* = 0.31) (Fig. 4C). RR less than 1.0 (for example, 0.81) means the event is less likely in the suture group compared with the screw group, and the *p*-value is 0.31, which is greater than 0.05, so it is not statistically significant. The range of motion was assessed as lag extension < 2° (79% versus 78%; RR 0.99 [0.90−1.09]; *p* = 0.84) and lag flexion < 5° (85% versus 87%; RR 1.03 [0.95−1.11]; *p* = 0.54) (Fig. 4D, E).

**Fig. 5:**
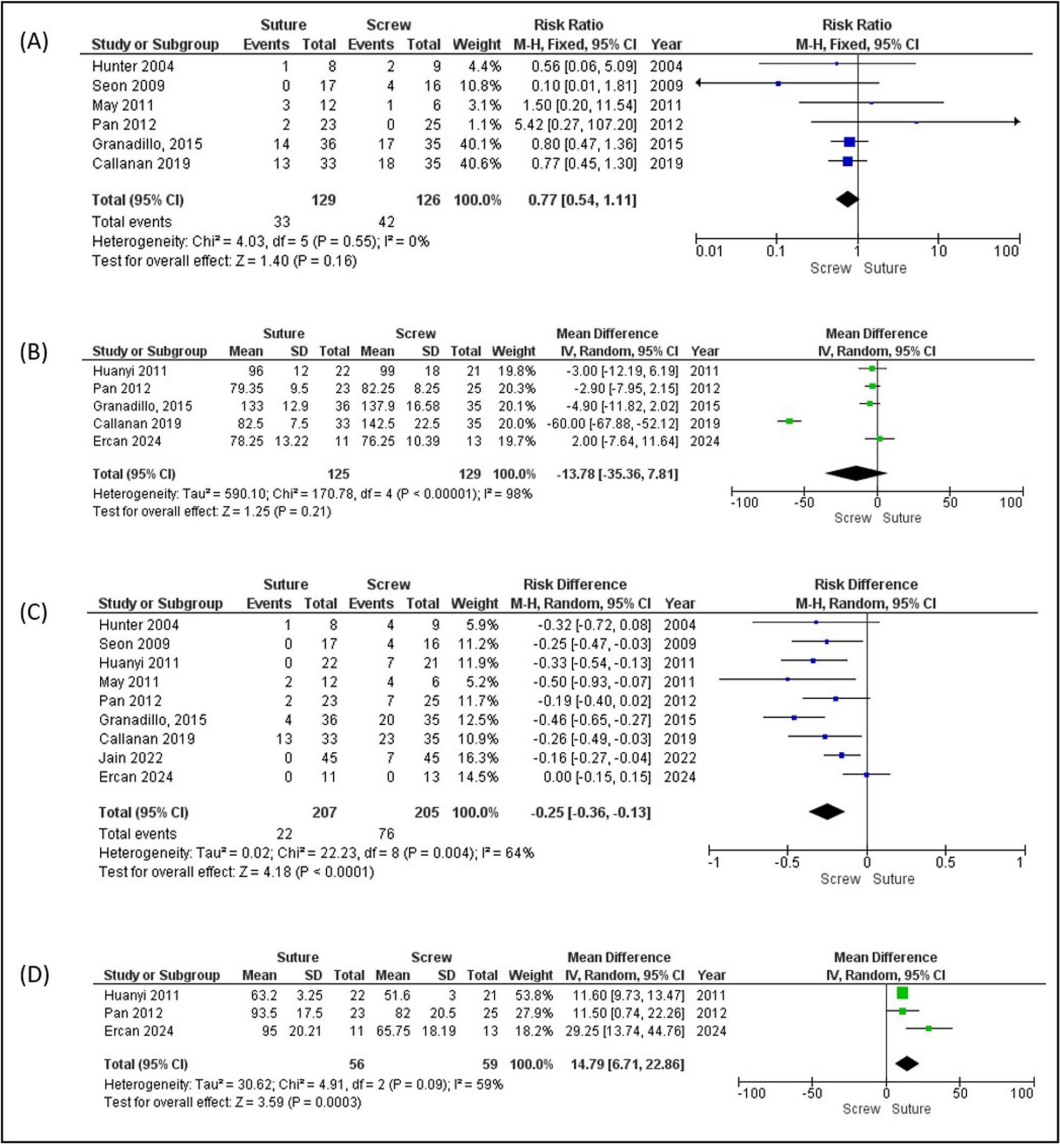
Forest plot analysis. **A** Complications, **B** union time, **C** subsequent surgery, and **D** duration of operation

Subgroup analysis revealed that the screw fixation group had a higher complication rate (32.2%) than the suture group (18.4%). This difference was statistically significant (risk difference (RD) −0.14 [−0.22 to −0.06]; *p* = 0.0003) (Fig. 5A). The suture group had a shorter union time (98 days versus 115 days), although this was not statistically significant (MD 13.78 [−35.36–7.81]; *p* = 0.21) (Fig. 5B). However, the screw fixation group had a significantly higher rate of subsequent surgery (29.75% versus 11.6%; RD −0.19 [−0.26 to −0.11]; *p* < 0.00001) and a shorter operation time (67 min versus 85 min; MD 14.79 [6.71–22.86]; *p* = 0.0003) compared with the suture fixation group (Fig. 5C, D).

**Fig. 6:**
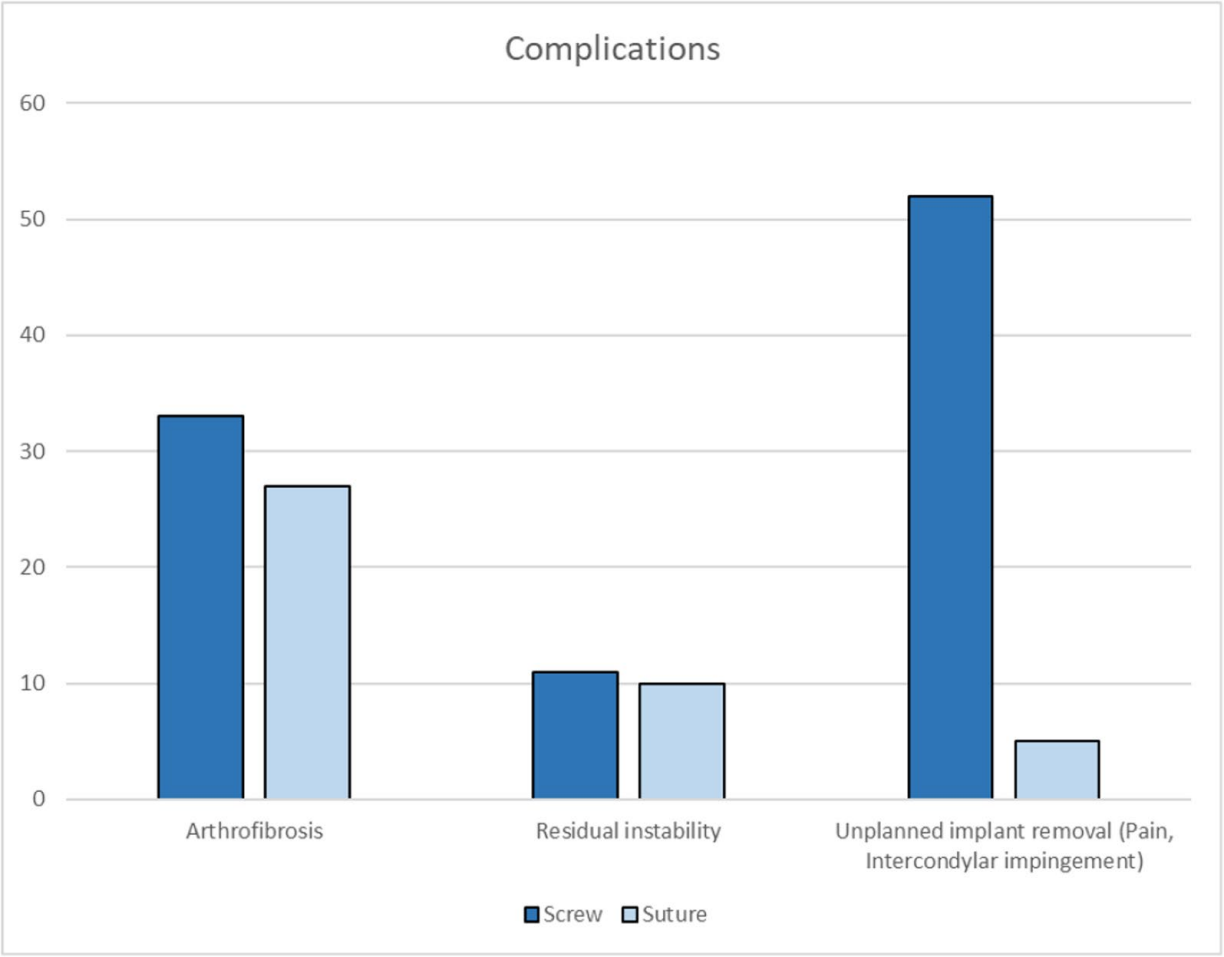
Complications after surgery

**Fig. 7:**
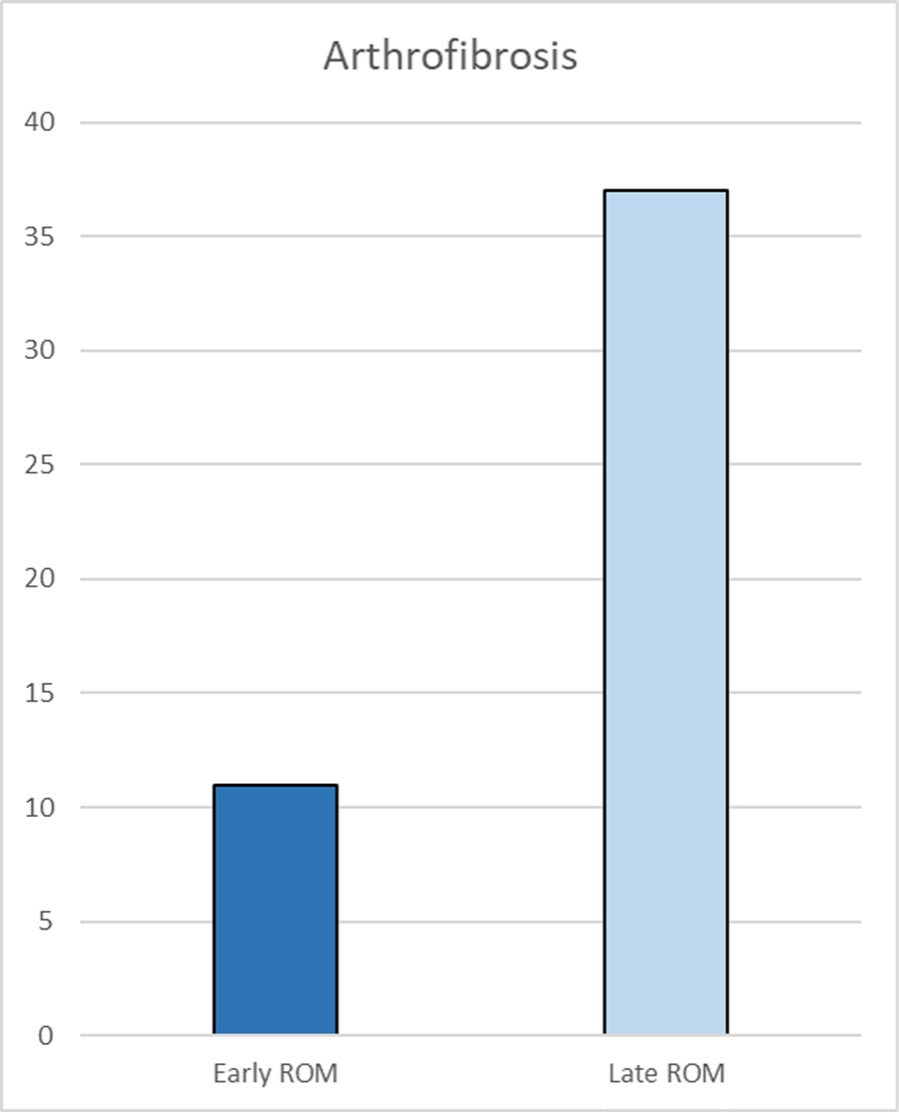
Arthrofibrosis incidence in early and late ROM exercise

**Fig. 8:**
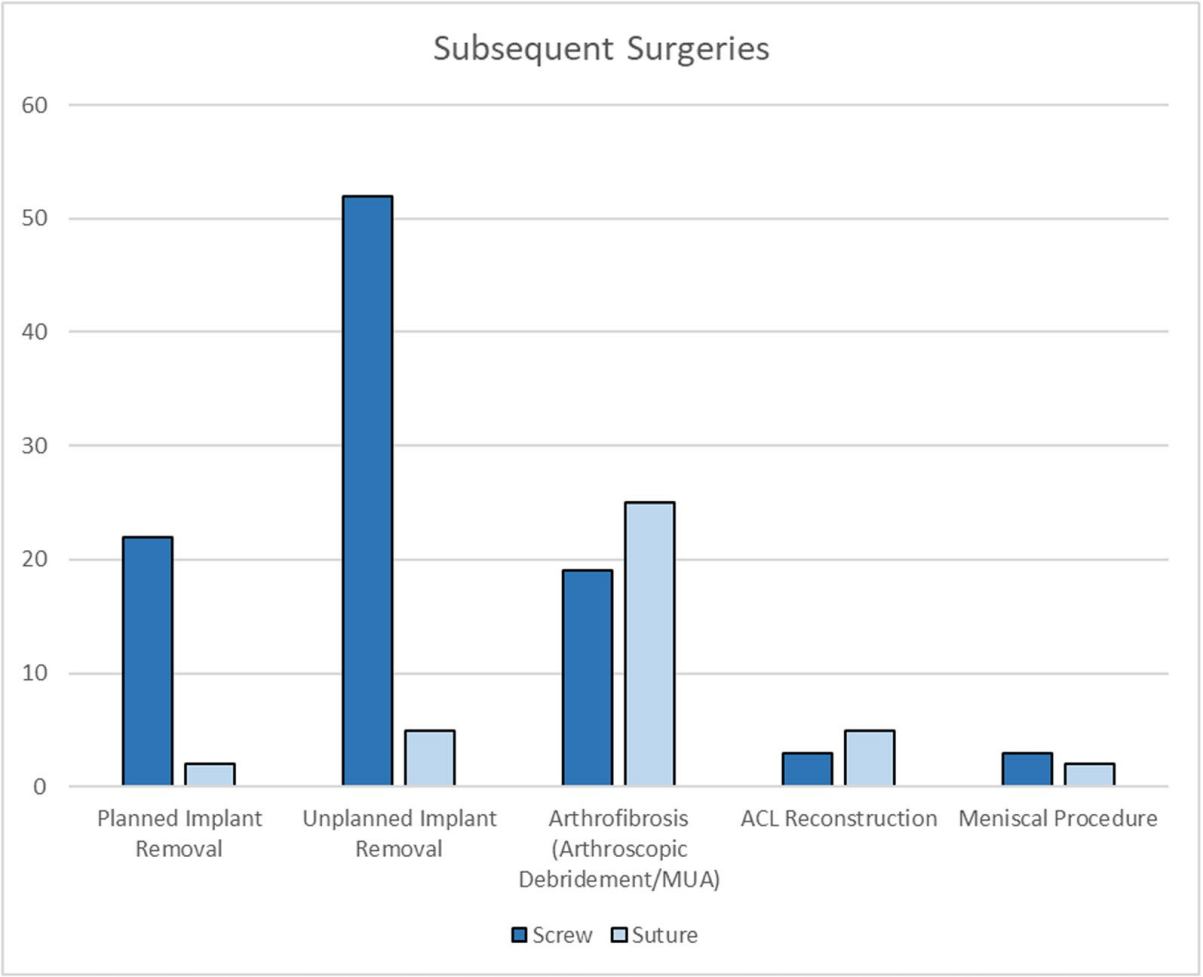
Subsequent surgeries after tibial eminence fracture fixation

The most common postoperative complication in suture fixation was arthrofibrosis (64.3%), while in the screw fixation group, it was unplanned implant removal (54.2%) (Fig. 6). There was no fracture displacement, delayed epiphyseal growth, and infection in the groups. Postoperative rehabilitation protocols varied across studies (Table 3). There was no significant correlation between arthrofibrosis and the timing of range of motion (ROM) initiation exercises (*p* = 0.404) (Figs. 7, 8).

**Table 3:** Rehabilitation program after surgery for tibial eminence fractures

Studies	Cast immobilization	Brace immobilization	Weight-bearing		Knee ROM	Guided physiotherapy		
			Partial	Full		Exercise	Running	Return to sports
Hunter, 2004	Not applicable	4 weeks (full extension)		Permitted as pain tolerated	Passive or active assisted ROM in prone position during first 4 weeks		After 8 weeks	After 12 weeks
Seon, 2009	3 weeks	4th–12th week	After 8 weeks	After 12 weeks	Start after 3 weeks	Quadriceps isometric exercise immediately after surgery		
May, 2011	3–6 weeks	2–6 weeks	Initially toe touch	After 4–8 weeks	Passive ROM immediately after surgery Passive ROM after 2 weeks			After 24 weeks
Huanyi, 2011	Not applicable	1 week (full extension)	After 4 weeks	After 8 weeks	Start after 2 weeks Reach 90° at 4th week	Straight leg raising on bed		
Pan, 2012	Not applicable	2 weeks (full extension)		Permitted as pain tolerated	0–90° during 3rd or 4th week 0–120° during 5th or 6th week	Quadriceps isometric exercise immediately after surgery	After 10 weeks	After 16 weeks
Granadillo, 2015	Not mentioned clearly in article							
Callanan, 2019	Not mentioned clearly in article							
Jain, 2022	Not applicable	2 weeks (full extension)	After surgery, with crutches	After 4 weeks		Patellar mobilization on 3rd day postoperation		After 24 weeks
Ercan, 2024	Not applicable	2 weeks (full extension)	After 4 weeks	After 6 weeks	0–90° during 3rd or 4th week 0–120° during 5th or 6th week	Quadriceps isometric exercise immediately after surgery		After 16 weeks

The most common subsequent surgery was implant removal, mainly due to unplanned implant removal (70.4%). Reoperation rate in suture group was mainly owing to arthroscopic debridement or manipulation under anesthesia due to arthrofibrosis (64.1%).

## Discussion

This systematic review and meta-analysis compared arthroscopic screw and suture fixation techniques for tibial eminence fractures, with a focus on clinical and functional outcomes. Our findings indicate that both methods were effective surgical options and offer satisfactory results, with no clear evidence favoring one approach over the other in terms of overall clinical success.

The functional outcomes of tibial eminence fracture treatment were evaluated using the Lysholm score, IKDC (both subjective and objective), and Tegner Activity Scale across multiple studies. In six studies, there was no statistical difference in the Lysholm score analysis between the screw and suture groups, which is consistent with prior findings. Most studies reported no difference in Lysholm scores between the two groups [4, 9–11]. However, Jain et al. (2023) reported better improvement in Lysholm scores in the suture group (mean difference = 33.16 versus 27.00) [12]. Similarly, subjective and objective IKDC scores showed no significant difference between the two techniques. Jain et al. (2023) noted a significant advantage for suture fixation in terms of subjective IKDC scores (MD 31.52 versus 26.38, *p* = 0.001) and Pan et al. (2012) reported better objective IKDC scores for screw fixation (*p* = 0.04) [12, 13]. Furthermore, age influenced IKDC results, with younger patients demonstrating superior outcomes [14]. Tegner Activity Scale evaluations also revealed no significant differences between fixation methods [9–11]. Collectively, these findings indicate that both screw and suture fixation are effective, with comparable functional outcomes.

Clinical outcomes following tibial eminence fracture fixation, assessed by KT-1000 arthrometer, Lachman test, pivot-shift test, and ROM, demonstrated no significant differences between arthroscopic screw and suture fixation. Lachman and pivot-shift tests showed comparable anterior and rotational stability (*p* = 0.95 and *p* = 0.7, respectively), although Pan et al. (2012) reported slightly better stability with screw fixation [13]. The KT-1000 arthrometer also demonstrated the results in line with the Lachman and pivot-shift tests (*p* = 0.31). While the screw fixation group had less anterior tibial translation than suture fixation (81% versus 62%), ROM deficits, including extension and flexion limitations, were also similar between groups (*p* = 0.84 and *p* = 0.54, respectively), likely influenced by postoperative immobilization protocols and rehabilitation adherence. Studies by Seon et al. (2009) and Callanan et al. (2019) supported these findings, reporting no significant differences in postoperative contracture or flexion limitations [4, 12, 15]. Collectively, these findings indicate that screw and suture fixation provide comparable clinical outcomes regarding ACL stability, anterior translation, and ROM recovery with minor variations in specific contexts.

Operative efficiency and fracture healing were evaluated by comparing surgical duration and union time between screw and suture fixation techniques. Across three studies, suture fixation was consistently associated with longer operative time (85 min versus 67 min), likely owing to the technical demands of soft tissue manipulation and knot-tying. However, the extended duration did not correlate with increased complication rates [4, 13]. In contrast, union time showed no significant difference between methods in most studies, with radiological healing typically observed by 3 months postoperatively [12, 16]. An exception was noted by Callanan et al. (2019), who reported longer union in the screw group (5.3 versus 3.2 months; *p* = 0.03) [15], possibly influenced by fracture complexity or imaging criteria for union. These findings suggest that screw fixation may offer procedural efficiency, which can reduce anesthesia time and resource utilization. Nevertheless, both techniques result in reliable union without elevated risks of infection or delayed healing.

Postoperative complications were significantly more frequent in the screw fixation group compared with the suture fixation group. The most prevalent complications included arthrofibrosis (43.5%), unplanned implant removal (41.3%), and residual instability (15.2%) (Fig. 6). The higher complication rate in screw fixation may be attributed to implant irritation due to hardware prominence, intra-articular hardware, and the need for more rigid fixation in comminuted fractures. Other complications, such as fracture displacement, delayed epiphyseal growth, infection, and traumatic arthritis, were not found during the follow-up period. Another study reported that common complications included reduced knee extension, pain during knee movement, instability, and arthrofibrosis, which occasionally require intervention [15, 17]. In addition, intermeniscal ligament obstruction has been noted as a potential barrier to proper reduction in some cases and may cause later complications [14, 17].

Arthrofibrosis, defined as a loss of ≥ 25° flexion and/or ≥ 10° extension compared with the contralateral knee, remains a notable complication after tibial eminence fracture fixation [18]. It is often attributed to prolonged immobilization and insufficient early ROM exercises. Early and appropriately paced rehabilitation is well documented to be critical in minimizing the risk of arthrofibrosis by promoting joint mobility and preventing excessive scar tissue formation [19]. In addition, delayed or overly aggressive rehabilitation can contribute to inflammation and fibrotic response, negatively impacting clinical outcomes [20]. Rehabilitation programs following surgery for tibial eminence fractures are crucial and vary across studies, typically involving immobilization for 3–4 weeks and gradual weight-bearing based on pain tolerance (Table 3) [4, 14, 21]. Extended immobilization (6–12 weeks) is anticipated to cause stiffness, muscle atrophy, and temporary joint damage [14]. However, this review showed no significant correlation between arthrofibrosis and the timing of ROM initiation exercises (Fig. 7). In addition, evidence suggests that early versus delayed weight-bearing strategies do not significantly impact functional scores or complication rates [21]. These findings underscore the need for individualized, balanced rehabilitation to optimize outcomes while minimizing complications.

Subsequent surgery refers to any procedure performed following tibial eminence fracture fixation associated with the initial surgery, often due to complications or the need for implant removal. In this study, only unplanned implant removal was included in statistical calculation. There was a significant difference in the analysis of subsequent surgeries between fixation methods, with a higher incidence in the screw fixation group. In this study, subsequent surgeries were mostly performed owing to unplanned implant removal (41%), arthrofibrosis (32%), and planned implant removal (17%) (Fig. 8). Most common causes of unplanned implant removal were pain and intercondylar impingement. Moreover, studies by Pan et al. (2012) and Callanan et al. (2019) reported ACL reconstruction and meniscal procedure as subsequent surgery following tibial eminence fixation [13, 15]. Additional procedures, including lysis of adhesions, manipulation under anesthesia, and arthroscopic debridement, were also more common in the screw fixation group [21]. These results emphasize that screw fixation is more likely to require additional surgery, mainly for implant removal.

Each screw and suture fixation offers distinct advantages and limitations in tibial eminence fractures. Screw fixation offers superior biomechanical stability, making it preferable for large or displaced fragments [10, 22]. Pan et al. reported that fixation with screws could be performed in patients with type IV ACL avulsion fractures [13]. However, it carries higher risks of implant-related complications, including prominence, growth plate injury in skeletally immature patients, and the need for hardware removal [17, 21, 23]. The use of low-profile or bioabsorbable screws may reduce such risks [9, 13, 14, 24]. In contrast, suture fixation is less invasive, minimizes physeal injury, results in less postoperative pain, and is effective for comminuted or smaller fragments but may result in longer operative times and slightly greater laxity [10, 25]. Although most laxity is clinically insignificant, suture outcomes are more technique-dependent, leading to variability in long-term stability [4, 14, 15, 26]. Both methods exhibit unique benefits and drawbacks, necessitating careful consideration of individual patient needs and fracture characteristics in surgical decision-making. 

## Limitations

This meta-analysis has several limitations. Firstly, the heterogeneity in age distribution and various age groups may have produced heterogeneous clinical outcomes. Secondly, there was variability in fracture type, rehabilitation protocols, and the time interval between injury and surgery. This heterogeneity may have led to the higher rate of bias in this study. Third, the retrospective nature of all the included studies may have introduced bias. Thus, it may be necessary to conduct randomized or prospective studies to evaluate the effectiveness of treatment for tibial eminence fractures. Fourth, only one cohort study with a larger sample size was included; the sample sizes of the other studies were small. Fifth, the included articles were predominantly of low-to-moderate risk of bias, which may affect the overall strength and reliability of the conclusions drawn. Sixth, our study could not regulate the fracture size, which may impact each fragment’s final fixation strength and the selected fixing method. Finally, subgroup and sensitivity analysis were not conducted in this study owing to heterogeneity of the data and limited reporting across included studies. Subgroup analyses based on age, fracture type, and rehabilitation protocols could be explored in future research to enhance the robustness and clinical applicability.

## Conclusions

The study found no notable difference in the clinical or functional outcomes between screw and suture fixation for tibial eminence fractures. However, suture fixation carried a significantly lower risk of subsequent surgery and complications but required a longer operation time than screw fixation. Both methods have advantages and disadvantages; therefore, the final decision must consider the patient profile and the specific clinical situation to achieve the best results.

## Data Availability

The datasets used and/or analyzed during the current study are available from the corresponding author on reasonable request.

## Abbreviations

PRISMA: Preferred Reporting Items for Systematic Reviews and Meta-Analyses
IKDC: International Knee Documentation Committee
ACL: Anterior cruciate ligament
ROB: Risk of bias
RCT: Randomized controlled trial
ROBINS-I: Risk of bias in nonrandomized studies of intervention
SPSS: Statistical Package for the Social Sciences
MD: Mean difference
RR: Risk ratio
RD: Risk difference
CI: Confidence interval

## References

[1] Axibal DP, Mitchell JJ, Mayo MH, Chahla J, Dean CS, Palmer CE et al (2019) Epidemiology of anterior tibial spine fractures in young patients: a retrospective cohort study of 122 cases. J Pediatr Orthopaed 39(2):E87-90 10.1097/BPO.0000000000001080 28945690

[2] Joshi A, Nagmani S, Basukala B, Kayastha N, Pradhan I (2019) Tibial spine avulsion of ACL. Injuries around the knee. Wolter Kluwer, pp. 22-44

[3] Yuan L, Shi R, Chen Z, Ding W, Tan H (2022) The most economical arthroscopic suture fixation for tibial intercondylar eminence avulsion fracture without any implant. J Orthop Surg Res 17(1):327 35752828 10.1186/s13018-022-03219-w PMC9233839

[4] Seon JK, Park SJ, Lee KB, Gadikota HR (2009) A clinical comparison of screw and suture fixation of anterior cruciate ligament tibial avulsion fractures. Am J Sports Med 37(12):2334–2339 19737989 10.1177/0363546509341031

[5] Anderson CN, Anderson AF (2011) Tibial eminence fractures. Clin Sports Med 30:727–742 22018313 10.1016/j.csm.2011.06.007

[6] Osti L, Buda M, Soldati F, Del Buono A, Osti R, Maffulli N (2016) Arthroscopic treatment of tibial eminence fracture: a systematic review of different fixation methods. Br Med Bull 118:73–90 27151952 10.1093/bmb/ldw018 PMC5127426

[7] Eggers AK, Becker C, Weimann A, Herbort M, Zantop T, Raschke MJ et al (2007) Biomechanical evaluation of different fixation methods for tibial eminence fractures. Am J Sports Med 35(3):404–411 17170161 10.1177/0363546506294677

[8] Hiraga Y, Toh S (2005) A biomechanical comparison of repair techniques for anterior cruciate ligament tibial avulsion fracture under cyclic loading. J Arthrosc Relat Surg 21(10):1197–1201 10.1016/j.arthro.2005.06.020 16226647

[9] Ercan N, Arıcan G, Şibar K, Özmeriç A, İltar S (2024) Clinical and functional outcomes of suture versus headless screw fixation for tibial eminence fractures in children. Am J Sports Med 52(4):948–955 38385198 10.1177/03635465241227440

[10] Chang CJ, Huang TC, Hoshino Y, Wang CH, Kuan FC, Su WR et al (2022) Functional outcomes and subsequent surgical procedures after arthroscopic suture versus screw fixation for ACL tibial avulsion fractures: a systematic review and meta-analysis. Orthop J Sports Med 10:1–8 10.1177/23259671221085945 PMC8990705 35400137

[11] Bogunovic L, Tarabichi M, Harris D, Wright R (2015) Treatment of tibial eminence fractures: a systematic review. J Knee Surg 28:255–262 25162406 10.1055/s-0034-1388657

[12] Jain S, Modi P, Dayma RL, Mishra S (2023) Clinical outcome of arthroscopic suture versus screw fixation in tibial avulsion of the anterior cruciate ligament in skeletally mature patients. J Orthop 1(35):7–12 10.1016/j.jor.2022.10.006 PMC9619313 36325248

[13] Pan RY, Yang JJ, Chang JH, Shen HC, Lin LC, Lian YT (2012) Clinical outcome of arthroscopic fixation of anterior tibial eminence avulsion fractures in skeletally mature patients: a comparison of suture and screw fixation technique. J Trauma Acute Care Surg 72(2):88 10.1097/TA.0b013e3182319d5a 22328000

[14] Hunter RE, Willis JA (2004) Arthroscopic fixation of avulsion fractures of the tibial eminence: technique and outcome. Arthroscopy 20(2):113–121 14760342 10.1016/j.arthro.2003.11.028

[15] Callanan M, Allen J, Flutie B, Tepolt F, Miller PE, Kramer D et al (2019) Suture versus screw fixation of tibial spine fractures in children and adolescents: a comparative study. Orthop J Sports Med 7(11):2325967119881961 31803786 10.1177/2325967119881961 PMC6876177

[16] Huang TW, Lee CY, Chen SY, Lin SJ, Hsu KY, Hsu RWW et al (2015) Outcomes and second-look arthroscopic evaluation after combined arthroscopic treatment of tibial plateau and tibial eminence avulsion fractures: a 5-year minimal follow-up orthopedics and biomechanics. BMC Musculoskelet Disord 16(1):1 26490156 10.1186/s12891-015-0769-x PMC4618521

[17] Huanyi L, Haishan W, Yuli W, Peiliang F, Yunli Z (2011) Effectiveness comparison of two arthroscopic different fixations for anterior cruciate ligament tibial eminence avulsion fractures. Chin J Repar Reconstr Surg 25:899 21923012

[18] Folkman MJ, Patel NM, Stevens AC, Cruz AI, Lee RJ, Kushare I et al (2024) To manipulate or not? Management of pediatric knee arthrofibrosis following operative fixation of tibial spine fractures. J Pediatr Orthop Soc N Am. 9:100122 10.1016/j.jposna.2024.100122 PMC12088325 40432684

[19] Shelbourne KD, Wilckens JH, Mollabashy A, Decarlo M (1991) Arthrofibrosis in acute anterior cruciate ligament reconstruction. The effect of timing of reconstruction and rehabilitation. Am J Sports Med. 10.1177/036354659101900402 1897645 10.1177/036354659101900402

[20] Manske RC, Prohaska D, Lucas B (2012) Recent advances following anterior cruciate ligament reconstruction: rehabilitation perspectives—Critical reviews in rehabilitation medicine. Curr Rev Musculoskelet Med 5(1):59–71 22249750 10.1007/s12178-011-9109-4 PMC3535126

[21] May JH, Levy BA, Guse D, Shah J, Stuart MJ, Dahm DL (2011) ACL tibial spine avulsion: mid-term outcomes and rehabilitation. Orthopedics. 10.3928/01477447-20101221-10 21323291 10.3928/01477447-20101221-10

[22] Gans I, Ganley TJ (2013) Tibial eminence fractures: a review and algorithm for treatment. Univ Pa Orthop J 23:20–23

[23] Granadillo VA (2015) Comparison of two fixation methods in treating displaced pediatric tibial eminence fractures at Boston Children’s Hospital. http://nrs.harvard.edu/urn-3:HUL.InstRepos:17295885. Accessed 7 Feb 2025

[24] Coyle C, Jagernauth S, Ramachandran M (2014) Tibial eminence fractures in the paediatric population: a systematic review. J Child Orthop 8(2):149–159 24585047 10.1007/s11832-014-0571-6 PMC3965767

[25] Senekovic V, Balazic M (2014) Bioabsorbable sutures versus screw fixation of displaced tibial eminence fractures: a biomechanical study. Eur J Orthop Surg Traumatol 24(2):209–216 23412317 10.1007/s00590-013-1176-3

[26] Gans I, Baldwin KD, Ganley TJ (2014) Treatment and management outcomes of tibial eminence fractures in pediatric patients: a systematic review. Am J Sports Med 42:1743–1750 24256714 10.1177/0363546513508538

